# YAEL: Your Advanced Electrode Localizer

**DOI:** 10.1523/ENEURO.0328-23.2023

**Published:** 2023-10-18

**Authors:** Zhengjia Wang, John F. Magnotti, Xiang Zhang, Michael S. Beauchamp

**Affiliations:** Department of Neurosurgery, Perelman School of Medicine, University of Pennsylvania, Philadelphia, PA 19104

## Abstract

Intracranial electroencephalography (iEEG) provides a unique opportunity to record and stimulate neuronal populations in the human brain. A key step in neuroscience inference from iEEG is localizing the electrodes relative to individual subject anatomy and identified regions in brain atlases. We describe a new software tool, Your Advanced Electrode Localizer (YAEL), that provides an integrated solution for every step of the electrode localization process. YAEL is compatible with all common data formats to provide an easy-to-use, drop-in replacement for problematic existing workflows that require users to grapple with multiple programs and interfaces. YAEL's automatic extrapolation and interpolation functions speed localization, especially important in patients with many implanted stereotactic (sEEG) electrode shafts. The graphical user interface is presented in a web browser for broad compatibility and includes an interactive 3D viewer for easier localization of nearby sEEG contacts. After localization is complete, users may enter or import data into YAEL’s 3D viewer to create publication-ready visualizations of electrodes and brain anatomy, including identified brain areas from atlases; the response to experimental tasks measured with iEEG; and clinical measures such as epileptiform activity or the results of electrical stimulation mapping. YAEL is free and open source and does not depend on any commercial software. Installation instructions for Mac, Windows, and Linux are available at https://yael.wiki.

## Visual Abstract

**Figure:**
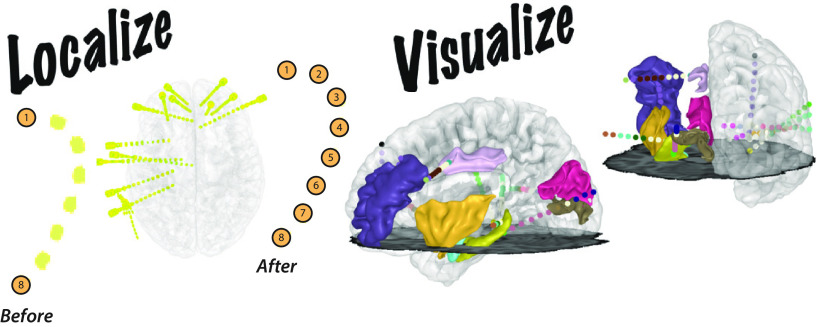

## Significance Statement

An essential step in the analysis of intracranial electroencephalography (iEEG) datasets is localizing the implanted electrodes relative to individual subject anatomy and identified regions in brain atlases. Your Advanced Electrode Localizer (YAEL) is a single, easy-to-use tool that accomplishes every step of the electrode localization process. YAEL is entirely integrated, unlike other solutions that require users to grapple with multiple programs and interfaces. Automatic extrapolation and interpolation functions speed localization, especially important in patients with many implanted stereotactic (sEEG) electrode shafts. The graphical user interface is presented in a web browser and includes an interactive 3D viewer for accurate localization of contacts on nearby shafts. After localization is complete, users may import data into the 3D viewer to create publication-ready visualizations.

## Introduction

Intracranial electroencephalography (iEEG) is a powerful technique in human neuroscience that records neural activity from electrodes implanted in the brain. A critical step in the analysis of iEEG data is defining the anatomic location of each electrode accurately and efficiently. Accurate electrode localization is critical for neuroscience inference, but efficiency is also important, as a single patient may be implanted with hundreds of electrodes.

The essential steps in electrode localization are straightforward. Typically, structural magnetic resonance imaging (MRI) scans are collected before the implantation surgery. After surgery, a computed tomography (CT) scan is obtained. The postoperative CT and preoperative MRI are then aligned. Electrode locations are identified using the CT (metal electrodes are easily localizable as high-intensity regions in the CT, but produce dark susceptibility artifacts in the MRI), then visualized on the MRI because of its superior anatomic contrast. Because of its importance, there are a plethora of tools and workflows for iEEG electrode localization, including SPM5/MATLAB (Hermes et al., 2010), iELVis (Groppe et al., 2017), ALICE (Branco et al., 2018), img_pipe (Hamilton et al., 2017), LeGUI (Davis et al., 2021), iEEGview (Li et al., 2019), BFM Tool (Wang et al., 2016), DELLO (Zhao et al., 2023), iElectrodes (Blenkmann et al., 2017), IELU (LaPlante et al., 2017), Slicer/CURRY (Trotta et al., 2018), EpiTools (Medina Villalon et al., 2018), FieldTrip (Stolk et al., 2018), and iEEG-recon (Lucas et al., 2023). There are also specialized tools for localizing deep brain stimulation (DBS) electrodes, such as Lead-DBS (Horn and Kühn, 2015; Horn et al., 2019), although in contrast with iEEG, DBS electrodes stimulate rather than record and are exclusively subcortical.

Given the numerous existing tools, what is the impetus for yet another electrode localizer? A major limitation of some existing methods is that they require scientists to install and learn several different software tools, written in different languages at different time by different groups using different data formats. Some packages, such as CURRY, are entirely commercial, charging thousands of dollars per license. Other software is freely available but relies on the commercial MATLAB package and add-on toolboxes purchased at additional expense. For example, before using iELVis, users must purchase a license and install MATLAB, then separately download and install the MATLAB routines in the legacy (unsupported) version of BioImage Suite (Papademetris et al., 2006), the MATLAB iELVis codebase, and MRIcroGL (Rorden and Brett, 2000). To localize electrodes, a complex sequence of steps in the different tools must be undertaken. YAEL streamlines the process so that users can perform all of the necessary steps within a single GUI that is free from reliance on commercial software.

A second limitation of some existing methods is that they are inefficient. Recently, clinical iEEG practice has transitioned from subdural electrodes, in which grids or strips are placed on the surface of the cortex [often referred to as electrocorticography (ECoG)], to stereotactic EEG (sEEG), in which many electrode shafts are inserted into the parenchyma. Although many older localization tools have features tailored to electrode grids, YAEL fully supports both subdural and sEEG electrodes. YAEL’s flexible 3D HTML WebGL-based brain viewer gives full control over dozens of visualization parameters. This control is especially important for sEEG, as it can be difficult to differentiate which electrodes belong to which shaft. To speed the localization process, YAEL provides automated tools for both interpolating and extrapolating electrode positions, removing the need to manually select all electrodes on an sEEG shaft or subdural grid or strip. Refinement tools ensure that manually selected electrode locations are located precisely at the center of the corresponding CT density.

**Figure 1.**
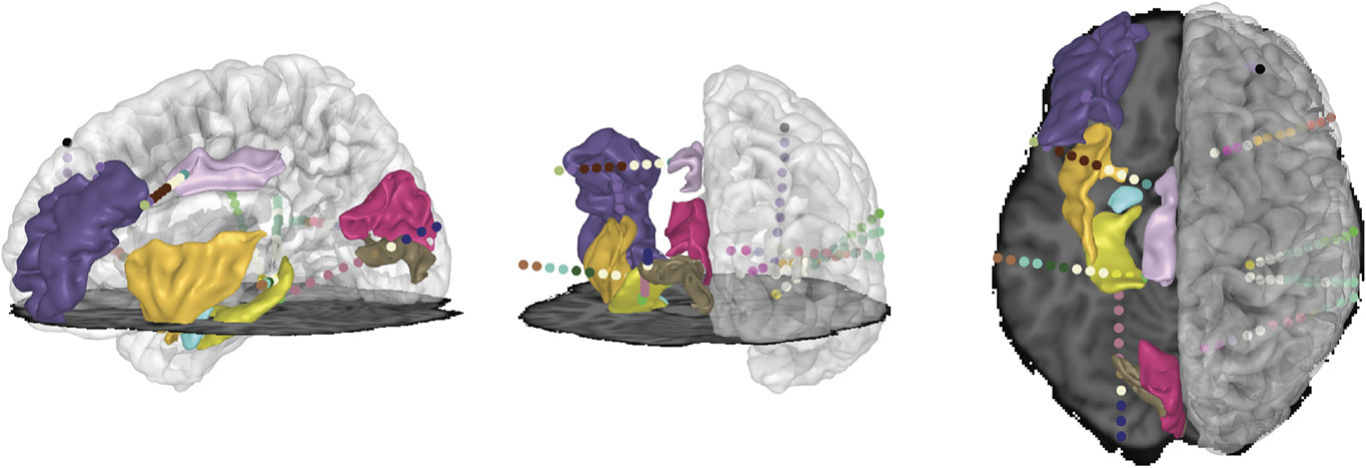
Highlights of the YAEL 3D viewer. Users can freely rotate the brain to show lateral view (left panel), posterior view (middle panel), or top view (right panel). Gray scale shows MRI data (horizontal plane shows axial slice through MRI data; right hemisphere shows translucent cortical surface model). Colored volumes show different anatomic regions of interest. Colored spheres show electrode contacts on different sEEG shafts. Color scale set by user and can reflect anatomic location and categorical or continuous experimental or clinical results (Fig. 4).

A third limitation of existing methods is that they offer limited utility: electrode locations must be exported to another package for further analysis and visualization. While YAEL can also function as a stand-alone localizer, a more powerful alternative is to use the flexible viewer in YAEL to make publication-quality images and movies using exactly the same GUI as for electrode localization. This flips the script on the traditional workflow, in which electrode locations are exported from the electrode localizer to another program. Instead, one of the many existing packages for iEEG data analysis, such as EEGLab (Delorme and Makeig, 2004), MNE-Python (Rockhill et al., 2022), FieldTrip (Oostenveld et al., 2011), RAVE (Magnotti et al., 2020), or a laboratory’s own software pipeline, can be used to calculate values for each electrodes. Then, these values are imported into YAEL for high-quality visualization. Figure 1 shows the outcome of this process, a combination of electrode, brain, and processed iEEG data combined with YAEL.

## Materials and Methods

### Code accessibility

Complete installation instructions for Mac, Windows, and Linux platforms, documentation and tutorial videos are available on the YAEL website, https://yael.wiki. The software download includes a sample dataset with a preimplant MRI and a postimplant CT. The source repository is available at https://github.com/beauchamplab/rave/. A Slack support channel is available to help new users get up to speed and resolve installation or usage problems.

## Results

**Figure 2.**
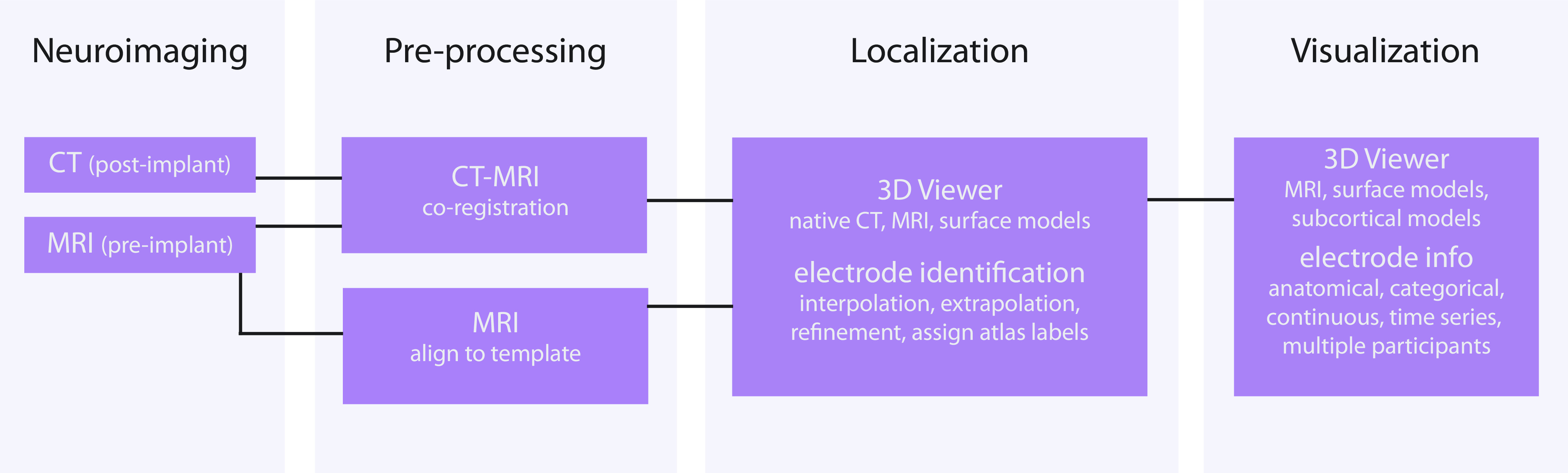
Flowchart of YAEL workflow.

Figure 2 provides an overview of the YAEL workflow, divided into the major steps in the workflow: image inputs; preprocessing; electrode localization; and data visualization. YAEL is designed to function using the computational resources available in any scientific laboratory and is built to be cross-platform, compatible with all recent versions of Windows, Mac, and Linux. The heart of YAEL is the 3D brain viewer, programmed in HTML and JavaScript, with WebGL enabling hardware acceleration. The brain viewer is incorporated into a GUI written in R (R Core Team, 2018) using shinyR (Chang et al., 2023) extensions. All user interactions occur through a web browser, ensuring a consistent user experience across platforms.

### Inputs

YAEL provides a file chooser to make it easy for users to specify the location of the required MRI and CT datasets, accepted in both NIFTI and DICOM formats. YAEL provides a form to enter an electrode plan specifying how the contacts are grouped into different sEEG shafts and ECoG grids or strips.

### Preprocessing

After identifying the imaging datasets, users select the desired tool for co-registration from a pull-down menu. YAEL includes two popular tools, Advanced Normalization Tools (ANTs; Tustison et al., 2021) and NiftyReg (Modat et al., 2014). For these tools, a single click within YAEL aligns the datasets. YAEL also supports one-click registration with FLIRT (Jenkinson et al., 2002; Greve and Fischl, 2009) for users that already have the extensive FMRIB software library (FSL) installed (Woolrich et al., 2009). The calls to external image registration routines are easily modifiable so that new tools, such as machine-learning registration algorithms (Iglesias, 2023) can be integrated into the workflow as additional items in the pull-down menu.

Parameters for the registration tool are set in the GUI and passed to the tool as command-line arguments. For instance, the default setting is rigid-body alignment (as this is faster and works well in most cases), but this can be modified to specify affine alignment. Comparing the multitude of techniques for cross-modal image registration is a complex endeavor and is beyond the scope of this manuscript, but for additional discussion see (Avants et al., 2011; Bartel et al., 2019; Iglesias, 2023).

Regardless of the registration tool and parameters selected, it is very important to verify the CT-MRI alignment by using the 3D viewer to examine the CT data overlaid on the MRI dataset. A common technique is to set a low threshold for the CT so that the skull is visible and ensure that the skull contour follows the scalp contour visible on MRI.

**Figure 3.**
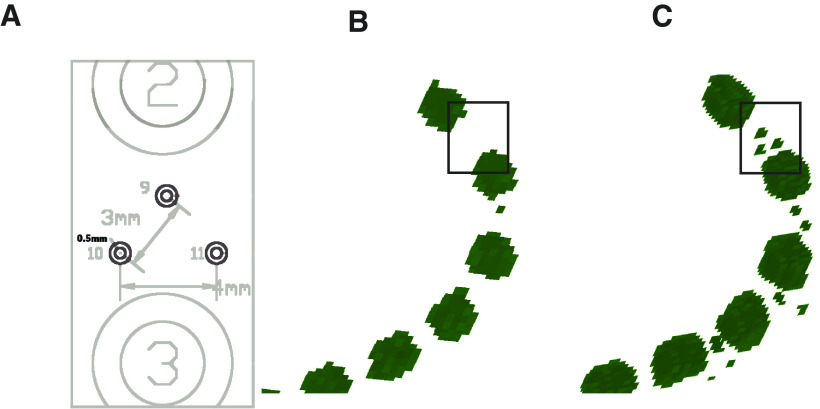
***A***, The manufacturer’s blueprint for an experimental subdural electrode strip, showing three miniature research contacts organized in a triangular pattern, located between two standard clinical contacts labeled “2” and “3.” ***B***, If the CT is downsampled to the MRI resolution before viewing, the three miniature research contacts are not visible (black rectangle). ***C***, In YAEL, the 3D viewer maintains the CT at the native resolution. The miniature research contacts are clearly visible (black rectangle).

Typical in-plane resolution is 0.3 mm for CT versus 1 mm for MRI. Down-sampling CT scans to MRI resolution can cause problems, such as the disappearance of smaller electrodes (Fig. 3). For this reason, YAEL uses the full-resolution CT during localization. Users may bypass co-registration if their CT/MRI has already been aligned, but should verify that the CT data were not down-sampled during alignment.

### Brain location identification

To identify the anatomic location of electrodes, YAEL supports aligning MRI data to a template brain. For instance, YAEL can use the included ANTs toolset to align the patient’s MRI data to the Montreal Neurologic Institute template (Evans et al., 1992). If users have created cortical surface models from the patient’s MRI, YAEL can load them for improved visualization.

To simplify surface model creation and anatomic identification, YAEL can call FreeSurfer with a single click (Dale et al., 1999; Fischl et al., 1999). FreeSurfer identifies each voxel in the patient’s MRI with an anatomic label, such as “corpus callosum,” “lateral occipital cortex,” “cerebrospinal fluid,” or “unknown” for out-of-brain (Fischl et al., 2004; Desikan et al., 2006; Destrieux et al., 2010). YAEL automatically transfers these anatomic labels to each electrode. A voting process is used to make labeling less sensitive to small electrode shifts: the most frequent label in a three-by-three-by-three cube of voxels centered on the electrode is determined and this label is assigned to the electrode (in the event of a tie, the label of the voxel at the cube’s center is used).

### Electrode localization

Identifying electrode locations is the most important and time-consuming aspect of the localization workflow, and YAEL provides several tools to improve the accuracy and efficiency of identifying electrodes. The most important tool is the sophisticated 3D viewer, which provides simultaneous visualization of 3D cortical surface models and 2D anatomic slices together with CT data and localized electrode positions. Users can rotate the brain using the mouse or keyboard shortcuts, adjust the transparency of cortical and subcortical surface models, and display any combination of axial, sagittal and coronal views of the MRI dataset (MRI is displayed in grayscale with an adjustable solid color overlay for the CT). With a 2D viewer, it can be difficult to determine which electrodes should be assigned to which shaft. In contrast, with YAEL’s 3D Viewer, the spatial orientation of the electrodes in the same shaft is immediately apparent. The viewer can be set to provide canonical axial, sagittal and coronal views, or “line-of-sight” views along the insertion trajectory of the sEEG shaft.

Double clicking in the vicinity of a CT density in either the 3D viewer or one of the 2D MRI slice views (axial, sagittal or coronal) creates a new electrode, visible as a sphere. This is different from most electrode localization workflows, in which location specification is only available in 2D. 3D specification is especially useful when nearby contacts are on different sEEG shafts.

YAEL applies an automatic refinement process so that sphere center (electrode location) is positioned at the center of the electrode, as determined by weighting the nearby CT densities. The location of the sphere can be manually adjusted with the mouse or keyboard for finer adjustment. As each electrode is localizer, YALE updates electrode table with 3D locations and atlas labels. A fully manual mode is also available, allowing users to simply select locations on the cortical surface or the MRI volume in situations where a CT is not available, such as for intraoperative iEEG cases.

### Automation

**Figure 4.**
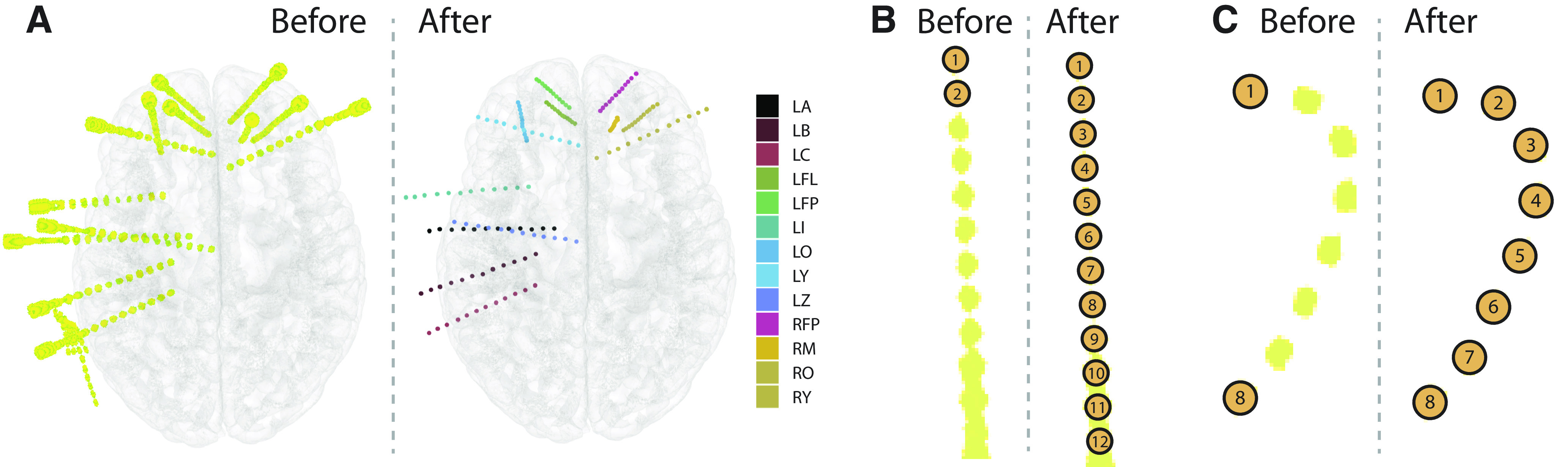
***A***, Before localization (left panel), the viewer allows flexible 3D manipulation and visualization of the CT dataset (yellow color) and the MRI dataset (transparent cortical surface models). After localization (right panel), each electrode contact is visible as a sphere, with contacts on each sEEG shaft in a different color (the legend shows the code assigned to each shaft during implantation surgery). ***B***, For automatic extrapolation, the user clicks the first two contacts of an sEEG shaft (shown) or a subdural electrode strip, then YAEL automatically localizes the remaining electrodes. ***C***, For automatic interpolation, the user clicks on the first and last contact on a subdural electrode strip (shown) or an sEEG shaft. Clicking the “interpolate” button automatically localizes the intermediate electrodes. Interpolation succeeds despite the sharp curvature generated as the subdural strip conforms to the occipital pole.

YAEL expedites the process of electrode localization with two automation tools. The first automation tool is extrapolation (Fig. 4*B*). The user selects the first two contacts in a shaft and clicks the “extrapolate” button, YAEL automatically determines the location of the intermediate contacts, populating the electrode table. For interpolation (Fig. 4*C*), the user selects two contacts at either end of an sEEG shaft, clicks the “interpolate” button, and then YAEL automatically determines all intermediate electrodes. The number of extrapolated or interpolated electrodes can be automatically determined from the electrode plan or specified manually.

To account for any bends in the electrode shaft, YAEL’s interpolation feature iteratively performs interpolate and extrapolate operations. The distance between the two most recently created contacts is measured to determine the likely location of the next contact, but because electrode arrays are not precisely linear (because of shaft bending for sEEG electrodes and curvature of the cortical surface for ECoG electrodes) the precise location often differs from the computed location, so YAEL sequentially examines nearby (<2 mm) CT voxels to identify the precise position of the contact (CT voxels near previously selected contacts are excluded to avoid duplicate selection). This iterative approach is effective, even with subdural strips that have substantial bends around the occipital pole, as shown in Figure 4*C*.

### Brain shift correction

Implantation of subdural electrodes typically requires a large craniotomy, which can lead to shifts in the location of the brain of 1 cm or more (Hermes et al., 2010); this shift does not occur for sEEG electrodes, which are inserted through small burr holes. To account for brain shift, YAEL implements the method described by (Hermes et al., 2010). Since subdural electrodes (by definition) sit on the brain surface, electrode locations in the postoperative CT that are inside the brain on the preoperative MRI must be there because of brain shift, so YAEL moves these electrodes to the nearest location on the brain surface of the preoperative MRI, as determined from the FreeSurfer pial envelope surface; electrode topology is preserved during the projection. Only contacts that are identified by the user as being of type ECoG in the electrode plan are subjected to the correction, and YAEL reports both the original and shifted electrode coordinates in the electrode table.

### Interoperability

YAEL is designed to easily interoperate with other software through data import and export. Electrode data are stored in a simple plain-text data table with one row per electrode and one column per field of data. Default YAEL fields include the electrode number, the channel label from the recording system, the co-ordinates of the electrode in native and standard spaces, and information about the anatomic assignment of the electrode. The BIDS-iEEG standard (Holdgraf et al., 2019) contains specifications for electrode metadata, with location in a tab-separated value file (e.g., sub-01_electrodes.tsv) and co-ordinate system in a JavaScript Object Notation file (e.g., sub-01_coordsystem.json). With a click, YAEL exports electrode information in both plain-text and BIDS-iEEG format to disk where it can be used for analysis or display in another package.

An alternative mode of YAEL usage is to import data about each electrode into YAEL, where it can be visualized using the 3D viewer. The user provides as many columns of data for each electrode as desired; YAEL intelligently uses the column label for visualization and ignores table cells with no data.

To make it easy for user to import data, YAEL automatically creates a template table with one row for each electrode which can be edited by the user with any text editor or spreadsheet software (such as Microsoft Excel). Data tables can also be imported from software packages specialized for analyzing iEEG voltage by time data, such as EEGLAB (Delorme and Makeig, 2004), FieldTrip (Oostenveld et al., 2011), EpiTools (Medina Villalon et al., 2018), MNE-Python (Rockhill et al., 2022), or RAVE (Magnotti et al., 2020).

To display data, YAEL assigns colors to each electrode based on a variety of user-selectable color scales. For more attractive visualization, the value at each electrode can also be mapped to nearby vertices of the brain surface model using the following formula:

vertexvalue=electrodevalue * exp(−distance * decay_factor/max_radius),

where *distance* is the distance between the electrode and the surface vertex, and no value is assigned for *distance* > *max_radius*. All parameters are user-selectable in the GUI, with defaults of *decay_factor* = 1.5 and *max_radius *=* *1 mm. In the event that a vertex is near multiple electrodes, the values are averaged weighted by *distance*. For discrete-valued datasets (where averaging would be inappropriate) YAEL assigns the value of the nearest electrode to all vertices with *distance* < *max_radius*.

### YAEL visualization

**Figure 5.**
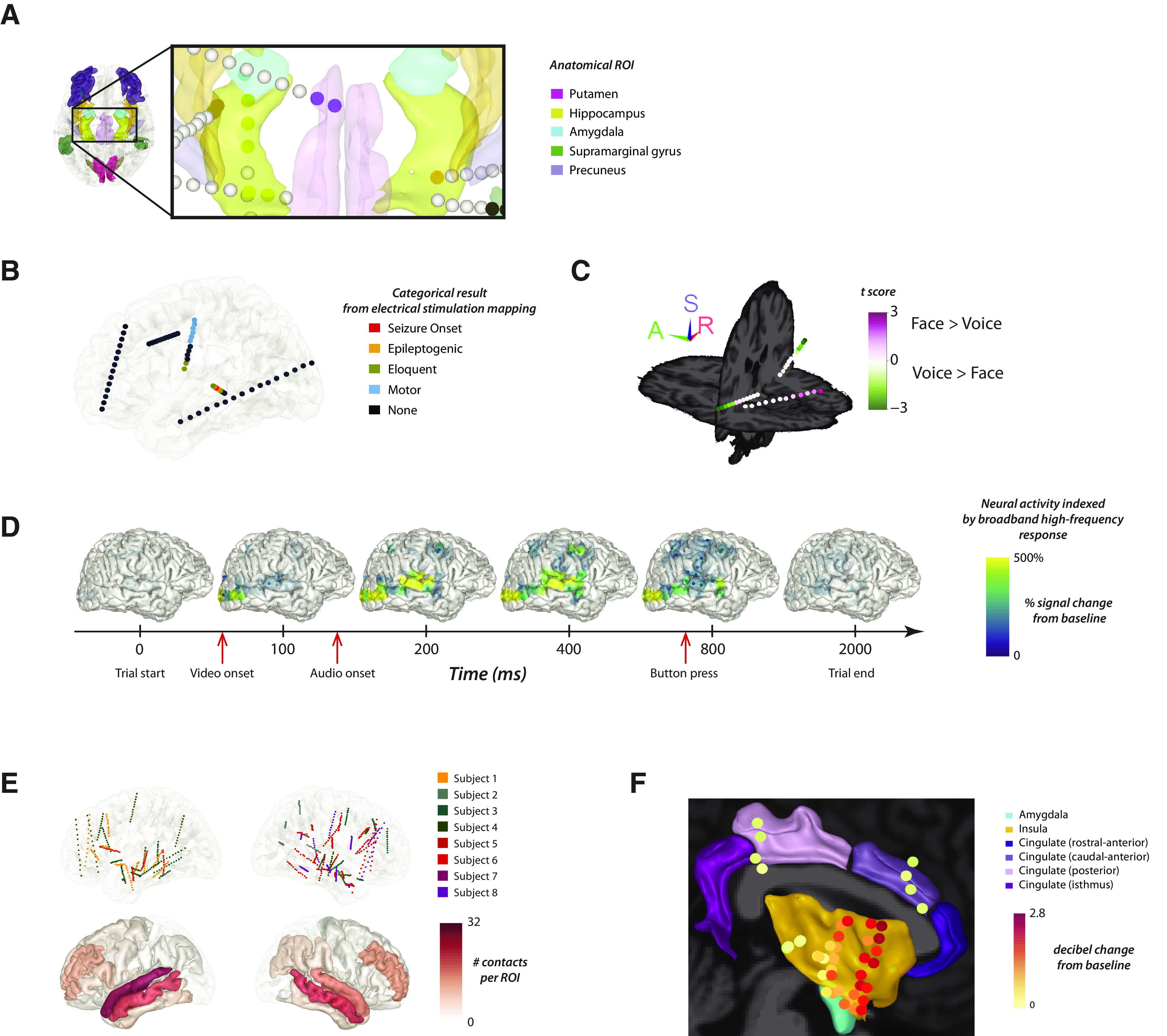
YAEL creates high-quality visualizations from a variety of iEEG data. ***A***, YAEL visualization of anatomic data. Colored volumes show different anatomic regions of interest (ROIs) from an atlas (legend at right). Spheres show sEEG electrode contacts. Colored contacts are located within an ROI, gray contacts are not in any ROI. ***B***, YAEL visualization of categorical data. Contacts are colored by the results of electrical stimulation mapping. ***C***, YAEL visualization of continuous data. Contacts are colored by the results of an analysis of iEEG power in two conditions, viewing faces and listening to voices. Only electrodes with a significant response are shown (nonsignificant contacts in gray). ***D***, YAEL visualization of timeseries data. Each brain shows the power at one time point. Still frames show a movie where activity in each electrode was projected to the cortical surface. Alternately, only electrode activity can be shown, without projection to the cortical surface. Full videos available on software website. ***E***, YAEL visualization of group data. Top row shows electrodes from multiple participants, visualized on a template brain (one color per participant). Bottom row shows summary data of number of contacts across all participants in each anatomic ROI. ***F***, YAEL visualization of combination data. Six anatomic regions were selected; all electrodes within each region across participants were selected; and then each electrode was colored by the iEEG response to a stimulus.

The usefulness of YAEL’s visualization capabilities is shown in five sample usage scenarios in Figure 5. In the first scenario, YAEL is used to visualize anatomic-functional information about each electrode available from brain atlases (Fig. 5*A*). YAEL automatically labels electrodes by their anatomic identification. Clicking on an electrode displays all the anatomic-functional information available about an electrode, along with the atlas the information is derived from. Users can highlight specific electrodes based on their anatomic atlas label. Users can select any combination of anatomic regions of interest (ROIs), and electrodes that do not fall in a desired ROI are colored gray. In this scenario, the user does not need to supply any additional information to YAEL because anatomic information is automatically populated in the electrode table during localization.

In the second scenario, YAEL is used to visualize categorical data about each electrode (Fig. 5*B*). For instance, in electrical stimulation mapping current is applied through the implanted electrodes, one at a time, and the effect documented in a categorical way (George et al., 2020). An electrode might receive a designation of “motor” (if a motor response was evoked by stimulation), “eloquent cortex” if language function was interrupted, or “epileptogenic zone” if epileptiform activity was triggered. To create the data, the template table created by YAEL can be edited (using Microsoft Excel or another editor) to insert the appropriate values for each electrode; YAEL ignores missing values so it is not necessary to supply complete data; for instance, come electrodes could be omitted from stimulation mapping and the “stimulation results” column in the table would be left blank for these electrode rows (in the viewer, electrodes with absent data are shown in gray).

In the third scenario, YAEL is used to visualize continuous data about each electrode, such as a clinical or research measure extracted from the iEEG data (Fig. 5*C*). With continuous measures such as power, a common display mode is to select a statistically-significant threshold where only values above the threshold are displayed; if above threshold, the electrode is colored according to the continuous value. YAEL allows the user to independently choose the threshold and the color scale to use. For instance, the power in a given frequency band (time-locked to performance of a task or presentation of a sensory stimulus) provides information about the functional specialization of brain areas. Power may either decrease or increase relative to baseline, so power in response to a sensory stimulus (such as viewing faces or listening to voices) can be colored with a cold-to-hot color scale in YAEL. To create these data, the program used to calculate the continuous measure saves the data to a table, which the user uploads to YAEL.

A fourth scenario is the display of time-series data (Fig. 5*D*). When provided with time-series data in the uploaded data table, YAEL can use them to create movies of brain activity over time (sample movies available on the YAEL website). For instance, while viewing a talking face, visual areas might respond first when the face become visible, followed by auditory areas at the onset of the talker’s voice (Karas et al., 2019; Metzger et al., 2020).

A fifth scenario is visualizing data across multiple participants (Fig. 5*E*). If electrodes from multiple individual participants have been localized, YAEL can align the brains to the same template and display all electrodes together on the template brain. This permits users to understand the anatomic distribution of electrodes across participants and the sample size available in each ROI.

It is also possible to combine the different scenarios (Fig. 5*F*). For instance, across all participants, electrodes in particular anatomic regions could be selected for display. Then, the response to an experimental task in those electrodes could be mapped to a continuous color scale, providing a concise, single figure that summarizes a large quantity of group data (Metzger et al., 2023). This also enables new discovery. For instance, the data plotted in Figure 5*F* reveals an anterior-to-posterior gradient within the insular cortex, where anterior electrodes respond more strongly than posterior electrodes. In traditional workflows, this might be missed: in many workflows, all electrodes within a single ROI are collapsed, and the iEEG data from different ROIs are shown in a summary plot (Sakon and Kahana, 2022). This is because traditional workflows often lack the ability provided by YAEL to easily visualize the power in each electrode separately.

## Discussion

There are a host of existing solutions for electrode localization, but YAEL offers some key benefits. YAEL incorporates into existing workflows easily by providing a single, integrated tool to complete all of the steps necessary for localization, in contrast with alternative methods that require multiple software tools and interfaces. The interactive 3D viewer allows users to ensure that even complex arrangements of sEEG electrode shafts are accurately localized, while automation tools expedite what can be a lengthy task. YAEL is designed with a modern, easy-to-use GUI, and all user interactions occur through a web browser. YAEL’s client/server architecture means that it can be installed on a desktop, a lab or university server, or in the cloud, and accessed by any number of users. Software installation is quick and does not require any commercial or licensed software such as MATLAB. Help with installation and usage is available from an active Slack support channel and in-person and remote tutorial sessions. YAEL’s functionality is not limited to electrode localization, as it allows users to import data for display in the 3D viewer. This enables the creation of publication-ready visualizations of common research and clinical iEEG data, including anatomic parcellation, categorical data, continuous data, time series data, and group data across participants.

## Synthesis

Reviewing Editor: William Stacey, University of Michigan Decisions are customarily a result of the Reviewing Editor and the peer reviewers coming together and discussing their recommendations until a consensus is reached. When revisions are invited, a fact-based synthesis statement explaining their decision and outlining what is needed to prepare a revision will be listed below. The following reviewer(s) agreed to reveal their identity: David Brang.

The goal of this work seeks to streamline several methods into one package as an integrated solution. There are many labs already doing this, but they tend to use homemade processes that string together several steps. The present tool offers some advantages over other published methods and may be appropriate for labs looking for an integrated solution. However, it does not include comparisons with other methods to demonstrate itself superior to these other methods. It might be helpful to add a brief description in the Discussion explaining why a lab might want to use this particular method.

One reviewer was able to install the software, but the other states that they tried to install/run but received the error Library/R/x86_64/4.2/library/raveio/R/raveio.rdb’ is corrupt. Please determine why this error occurred and correct it.

Several options are discussed for each stage of registration. No validation data is presented showing that the default parameters used result in more accurate registration at the single-subject or group-level.

Both reviewers independently noted a significant issue that needs to be discussed. It is unclear if subdural electrode registration includes measures to counter the effect of post-operative shift of the brain, resulting in electrode locations on the CT that can appear to be inside of the brain (on the T1). Other programs differ in using a nearest neighbor approximation or by using the shape of the electrode to counteract the apparent drift. Perhaps this was simply not mentioned in the manuscript, as Figure 4D indicates that there is an option to project activity from each electrode to the cortical surface. The article should state which algorithm is used for projecting both electrodes and activity to the cortical surface.

When projecting activity to the cortical surface in a smooth map, is there any correction for electrode density applied? In case electrodes are spaced more densely in certain areas compared to others, not including a correction can lead to inadvertently concluding that there is an increase in activity.

Is there an option to export relevant data in BIDS format without manually recoding the outputs?

Does the program label tissue type or whether electrodes are outside of the brain

Additional information on how anatomical regions are assigned would be beneficial; e.g., if an electrode is in white matter adjacent to two cortical regions (or between two gyri), how is it given a single assignment?

Minor comments

On page 6, line 125, the manuscript text “2D anatomical slices together with CT data and localized electrode positions (Figure 3A)” seems to indicate that Figure 3A shows anatomical slices with CT data, but that appears to only be in Figure 4C. Please add to the figure, or correct text accordingly.

Figure 2. The labels appear flipped. CT is currently labeled pre-implant and MRI post-implant.

In the sentence citing previous iEEG electrodes localization tools (“Because of its importance, there are a plethora of tools for iEEG electrode localization, including ...”), there are some additional citations that should be added:

- Fieldtrip: Stolk, A., Griffin, S., van der Meij, R. et al. Integrated analysis of anatomical and electrophysiological human intracranial data. Nat Protoc 13, 1699-1723 (2018). https://doi.org/10.1038/s41596-018-0009-6

- ctmr: Hermes, D., Miller, K. J., Noordmans, H. J., Vansteensel, M. J., & Ramsey, N. F. (2010). Automated electrocorticographic electrode localization on individually rendered brain surfaces. Journal of neuroscience methods, 185(2), 293-298.

## Author Response

Authors’ Rebuttal

We are grateful for the reviewers’ suggestions and the editor’s synthesis and have changed the manuscript to completely address them, as detailed below.

1. Extended Data should be labeled as Figure 1-1, Figure 1-2, Table 1-1, etc., so they indicate which figure they are supporting (i.e. Extended Data table supporting Figure 5 labeled as Figure 5-1). This change is not necessary at this stage, but will be if your paper receives a revise ¬decision.

R1. We have renamed “Supplementary Figure 1” to “Figure 3”.

2. The goal of this work seeks to streamline several methods into one package as an integrated solution. There are many labs already doing this, but they tend to use homemade processes that string together several steps. The present tool offers some advantages over other published methods and may be appropriate for labs looking for an integrated solution. However, it does not include comparisons with other methods to demonstrate itself superior to these other methods. It might be helpful to add a brief description in the Discussion explaining why a lab might want to use this particular method.

R2. Thank you for this helpful suggestion, we now write:

“There are a host of existing solutions for electrode localization, but YAEL offers some key benefits. YAEL incorporates into existing workflows easily by providing a single, integrated tool to complete all of the steps necessary for localization, in contrast with alternative methods that require multiple software tools and interfaces. The interactive 3D viewer allows users to ensure that even complex arrangements of sEEG electrode shafts are accurately localized, while automation tools expedite what can be a lengthy task. YAEL is designed with a modern, easy-to-use GUI, and all user interactions occur through a web browser. This client/server architecture means that YAEL can be installed on a desktop, a lab or university server, or in the cloud, and accessed by any number of users. Software installation is quick and does not require any commercial software such as Matlab. Help with installation and usage is available from an active Slack support channel and in-person and remote tutorial sessions. YAEL’s functionality is not limited to electrode localization, as it allows users to import data for display in the 3D viewer. This enables the creation of publication-ready visualizations of common research and clinical iEEG data, including anatomical parcellation, categorical data, continuous data, time series data, and group data across participants.”

3. One reviewer was able to install the software, but the other states that they tried to install/run but received the error Library/R/x86_64/4.2/library/raveio/R/raveio.rdb’ is corrupt. Please determine why this error occurred and correct it.

R3. Our guess is that reviewer updated the software but did not restart R. We now emphasize the importance of this step in the Troubleshooting section of the Installation instructions (at http://yael.wiki):

Many common problems, such as the error Library/R/x86_64/4.2/library/raveio/R/raveio.rdb is corrupt can be resolved by quitting and restarting RStudio.

4. Several options are discussed for each stage of registration. No validation data is presented showing that the default parameters used result in more accurate registration at the single-subject or group-level.

R4. This is a very complex topic and we now write in the manuscript:

There are many options for image registration, and YAEL includes two popular choices, Advanced Normalization Tools (ANTs) (Tustison et al., 2021) and NiftyReg (Modat et al., 2014). For these tools, a single click within YAEL aligns the datasets. YAEL also supports one-click registration with FLIRT (Greve and Fischl, 2009; Jenkinson et al., 2002) for users that already have the extensive FMRIB software library (FSL) installed (Woolrich et al., 2009). The calls to external image registration routines are easily modifiable so that new tools, such as machine-learning registration algorithms (Iglesias, 2023) can be integrated into the workflow as additional items in the pull-down menu.

By default, rigid-body registration is used because it is faster and usually gives reasonable results. However, the command line arguments provided to the registration tool can be modified from within the GUI in order to, for example, select affine instead of rigid-body registration. Comparing techniques for cross-modal image registration is a complex endeavor and is beyond the scope of this manuscript, but for additional discussion see (Avants et al., 2011; Bartel et al., 2019; Iglesias, 2023).

Regardless of the registration tool and parameters selected, it is very important to verify the CT-MRI alignment by using the 3D viewer to examine the CT data overlaid on the MRI dataset. A common technique is to set a low threshold for the CT so that the skull (as well as the electrodes) are visible and ensure that the skull contour follows the scalp contour visible on MRI.

5. Both reviewers independently noted a significant issue that needs to be discussed. It is unclear if subdural electrode registration includes measures to counter the effect of post-operative shift of the brain, resulting in electrode locations on the CT that can appear to be inside of the brain (on the T1). Other programs differ in using a nearest neighbor approximation or by using the shape of the electrode to counteract the apparent drift. Perhaps this was simply not mentioned in the manuscript, as Figure 4D indicates that there is an option to project activity from each electrode to the cortical surface. The article should state which algorithm is used for projecting both electrodes and activity to the cortical surface.

R5. This is an important point, and we now write (see also response to next query):

Brain Shift Correction

Implantation of subdural electrodes typically requires a large craniotomy, which can lead to shifts in the location of the brain of 1 cm or more (Hermes et al., 2010); this shift does not occur for sEEG electrodes, which are inserted through small burr holes. To account for brain shift, YAEL implements the method described by (Hermes et al., 2010). Since subdural electrodes by definition sit on the brain surface, locations inside the brain are attributable to brain shift, so YAEL moves these electrodes to the nearest location on the brain surface, as determined from the FreeSurfer pial envelope surface. Electrode topology is preserved during the projection. Only contacts that are identified by the user as being of type ECoG (subdural) are subjected to the correction. YAEL reports both the original and shifted electrode coordinates in the electrode table.

6. When projecting activity to the cortical surface in a smooth map, is there any correction for electrode density applied? In case electrodes are spaced more densely in certain areas compared to others, not including a correction can lead to inadvertently concluding that there is an increase in activity.

R6. We now describe the algorithm in detail to make it clear that densely spaced electrodes are accounted for:

To display data, YAEL assigns colors to each electrode based on a variety of user-selectable color scales. Given the small surface area of each electrode, it can be difficult for users to discern electrode colors. To increase visibility, electrode values can be mapped to the cortical surface using the formula vertex value = electrode value * exp(- distance * decay_factor / max_radius) where distance is the distance between the electrode and the surface vertex, and no value is assigned for distance > max_radius. All parameters are user-selectable in the GUI, with defaults of decay_factor = 1.5 and max_radius = 1 mm. In the event that a vertex is near multiple electrodes, the values are averaged weighted by distance. For discrete-valued datasets (where averaging would be inappropriate) YAEL assigns the value of the nearest electrode to all vertices with distance < max_radius.

7. Is there an option to export relevant data in BIDS format without manually recoding the outputs?

R7. This is an excellent suggestion and we have added this option to the software. The update is described in the manuscript:

Electrode data is stored in a simple plain-text data table with one row per electrode and one column per field of data. Default YAEL fields include the electrode number, the channel label from the recording system, the co-ordinates of the electrode in native and standard spaces, and information about the anatomical assignment of the electrode. The BIDS-iEEG standard (Holdgraf et al., 2019) contains specifications for electrode metadata, with location in a tab-separated value file (e.g. sub-01_electrodes.tsv) and co-ordinate system in a JavaScript Object Notation file (e.g. sub-01_coordsystem.json). With a click, YAEL exports electrode information in both plain-text and BIDS-iEEG format to disk where it can be used for analysis or display in another package.

8. Does the program label tissue type or whether electrodes are outside of the brain. Additional information on how anatomical regions are assigned would be beneficial; e.g., if an electrode is in white matter adjacent to two cortical regions (or between two gyri), how is it given a single assignment?

R8. We now clarify as follows:

FreeSurfer identifies each voxel in the patient’s MRI with an anatomical label, such as “corpus callosum”, “lateral occipital cortex”, “cerebrospinal fluid” or “unknown” for out-of-brain (Desikan et al., 2006; Destrieux et al., 2010; Fischl et al., 2004). YAEL automatically transfers these anatomical labels to each electrode. There is not a one-to-one correspondence between electrode locations (which are defined by a single point) and voxels (which are a volume) so a voting process is used to make labelling less sensitive to small positional shifts. The most frequent label (other than “unknown” or “white matter”) in a three-by-three-by-three cube of voxels centered on the electrode is determined and this label is assigned to the electrode. In the event of a tie, the label of the voxel at the cube’s center is used. All labels are written as text to the electrode table where they can be hand-edited if desired. The user also can manually create additional column(s) to list other anatomical identifications.

Minor comments

9. On page 6, line 125, the manuscript text “2D anatomical slices together with CT data and localized electrode positions (Figure 3A)” seems to indicate that Figure 3A shows anatomical slices with CT data, but that appears to only be in Figure 4C. Please add to the figure, or correct text accordingly.

R9. We have removed the figure reference to avoid confusion.

10. Figure 2. The labels appear flipped. CT is currently labeled pre-implant and MRI post-implant.

R10. The error has been corrected.

11. In the sentence citing previous iEEG electrodes localization tools (“Because of its importance, there are a plethora of tools for iEEG electrode localization, including ...”), there are some additional citations that should be added: - Fieldtrip: Stolk, A., Griffin, S., van der Meij, R. et al. Integrated analysis of anatomical and electrophysiological human intracranial data. Nat Protoc 13, 1699-1723 (2018). https://doi.org/10.1038/s41596-018-0009-6 - ctmr: Hermes, D., Miller, K. J., Noordmans, H. J., Vansteensel, M. J., & Ramsey, N. F. (2010). Automated electrocorticographic electrode localization on individually rendered brain surfaces. Journal of neuroscience methods, 185(2), 293-298.

R11. The additional citations (and a reference to Trotta et al.) have been added at the suggested location. We thank the reviewer for pointing out the omission.
